# Supplementary material for: Management compliance attention, social performance and pay gap: Evidence from China
Source: PLoS One. 2025 May 27;20(5):e0324031. doi: 10.1371/journal.pone.0324031 (PMC12112162; doi:10.1371/journal.pone.0324031)
Supplement: Attachment — Submitted filename: Response to Reviewers.docx [file pone.0324031.s004.docx]

Response to Reviewer Comments

We sincerely appreciate the reviewer’s constructive feedback, which has significantly improved the clarity and rigor of our manuscript. Below is our point-by-point response to each comment:

1. Methodology Weakness in Introduction and Abstract

Abstract: We have expanded the abstract to include key results with metric figures (e.g., β coefficients, significance levels) and explicitly link findings to the theoretical framework. For example, we now state: “Management compliance attention is negatively correlated with pay gap (β = -0.010, p < 0.01), and social performance strengthens this negative effect (β = -0.022, p < 0.05).”

Introduction: We have revised the introduction to explicitly outline the study’s components, variables, and relationships. Specifically, we introduces the textual analysis of annual reports for measuring management compliance attention, the Huazheng ESG ratings for social performance, and the models used to test hypotheses.

2. Lack of Clarity on Study Novelty and Aim

Introduction: We have revised the introduction to explicitly state the research objective: “This study investigates how management compliance attention and social performance interact to mitigate pay gaps, advancing behavioral and equity theories in the context of China’s evolving regulatory environment.”

We highlight the novelty in the theoretical framework (Introduction), emphasizing how integrating behavioral theory (managerial attention) and equity theory (distributive justice) provides a unique lens to explain compliance’s role in pay equity. Additionally, we stress the originality of using textual analysis to operationalize compliance attention, a method underutilized in prior literature.

3. Grammatical Errors and English Proofreading

We focused on grammatical correctness, clarity, and adherence to academic writing conventions. Key improvements include refining sentence structure, ensuring consistent terminology, and enhancing flow in theoretical discussions.

4. Abstract Insufficiently Supported by Data

The revised abstract now includes specific statistical findings (e.g., β coefficients, significance levels) and directly links them to the research questions. For example:

“Results show that management compliance attention reduces pay gap (β = -0.010, p < 0.01), and social performance strengthens this effect (β = -0.022, p < 0.05). Organizational resilience partially mediates this relationship (Sobel test z = -3.02, p = 0.003).”

Additionally, in the discussion (Section 6), we explicitly connect the mean pay gap (2.68x) to compliance mechanisms, explaining how transparency and fairness norms under compliance reduce disparities.

5. Theoretical Incoherence and Mixing Theories

Literature Review: We streamlined the theoretical foundation to focus on behavioral theory (managerial attention and cognitive limitations) and equity theory (distributive justice). We explicitly link these theories to the research hypotheses and empirical analysis, demonstrating how compliance attention aligns with procedural fairness (behavioral theory) and social performance reinforces equity norms (equity theory).

Theoretical Framework Figure: We drew Figure 1 to visually emphasize the integration of these two theories, clarifying their complementary roles in explaining the compliance-pay gap relationship.

6. Weak Theoretical Contribution and Peripheral Theme

We have strengthened the theoretical contribution in Section 7:

Behavioral Theory Extension: We elaborate on how compliance attention operationalizes the attention-based view, showing how managerial focus on compliance signals fairness and reduces pay gaps.

Equity Theory Application: We discuss how social performance moderates compliance’s effect by amplifying stakeholder pressure for equity, extending equity theory into CSR contexts.

Organizational Resilience Mediator: We highlight how resilience bridges compliance to pay equity, advancing dynamic capabilities theory.

These revisions ensure the study contributes uniquely to the literature on compliance, social performance, and pay equity. The sections we have revised with major changes are highlighted in red.
