# Supplementary material for: Management compliance attention, social performance and pay gap: Evidence from China
Source: PLoS One. 2025 May 27;20(5):e0324031. doi: 10.1371/journal.pone.0324031 (PMC12112162; doi:10.1371/journal.pone.0324031)
Supplement: Attachment — Submitted filename: Reviewer Comments.docx [file pone.0324031.s002.docx]

Comments

The study addresses important issues of Management compliance attention and pay gap: Evidence from China and contributes knowledge to debates on factors affecting pay gap. Unfortunately;

1. One essential aspect of the study is only weakly addressed in the introduction and not followed up in the study itself: the methodology. I expected components, variables and relationship and how these correlate in the abstract section, but the paper does not offer a clear conclusion of the results and findings from the discussion. Your abstract should summarize the findings with the various components from your discussions

2. The authors are not able to demonstrate the novelty of this results? What is the ultimate aim of this study? This is not clearly stated and it is confusing.

3. Also, the manuscript need to be proof read by a native English person to correct the niggling grammatical errors.

4. The abstract summarizes the study objectives, the results, findings and recommendations: However, the author on stated that “The research shows that management compliance attention is negatively correlated with pay gap, and that social performance strengthens the negative effect of management”. These are general without any metric figures that justifies that, compliance is negatively correlated. This study is poorly presented, not far from narration of a story but I suggest the authors can improve upon this and brings out the novelty in this study. For instance the authors reported that, the mean value of Pay_gap is 0.987, with a standard deviation of 0.653, indicating substantial differences in the pay gap. So how does this relates to management compliance and how can this be addressed?

5. The authors mentioned a number of theories, such as the equity theory, echelon theory, Behavioral theories, the relative deprivation theory which are good for the analysis of this study especially the Behavioral theory but failed to nail their review and analysis on one or two theory and demonstrate how it best fit this study. Currently, the authors are mixing up three or more theories together without demonstrating how these relates best to management compliance. Therefore, the coherency is missing.

6. Finally, the theme is peripheral and opens a parallel discussion that do not contribute enough to define, explain and understand the relationship between the key concepts used. I think the paper is very weak to be published in this Journal and I think the paper is lacking in-depth theoretical discussions. This study should not be accepted for publication in this journal in its current form.
